# Identifying priority interventions for stroke in Ireland through stakeholder engagement to inform population-based modelling: a mixed methods protocol

**DOI:** 10.12688/hrbopenres.13413.1

**Published:** 2021-09-30

**Authors:** Eithne Sexton, Anne Hickey, David J. Williams, F. Horgan, Elaine Byrne, Chris Macey, Padraic Cuffe, Suzanne Timmons, Rónán Collins, K. Bennett

**Affiliations:** 1 Division of Population Health Sciences, Royal College of Surgeons in Ireland, Dublin, Ireland; 2 Department of Geriatric and Stroke Medicine, RCSI University of Medicine and Health Sciences, Dublin, Ireland; 3 Department of Geriatric and Stroke Medicine, Beaumont Hospital, Dublin, Ireland; 4 School of Physiotherapy, RCSI University of Medicine and Health Sciences, Dublin, Ireland; 5 Graduate School of Healthcare Management, RCSI University of Medicine and Health Sciences, Dublin, Ireland; 6 Irish Heart Foundation, Dublin, Ireland; 7 Patient collaborator, Sligo, Ireland; 8 Centre for Gerontology and Rehabilitation, University College Cork, Cork, Ireland; 9 National Dementia Office, Health Service Executive, Tullamore, Co Offaly, Ireland; 10 National Clinical Programme for Stroke, Health Service Executive, Dublin, Ireland; 11 Age-Related Health Care and Stroke Service, Tallaght University Hospital, Dublin, Ireland

**Keywords:** Stroke, mixed methods, stakeholder engagement, policy, intervention, priority setting

## Abstract

**Introduction**

Improvements in stroke survival have resulted in increasing numbers of people living with stroke, and with a rapidly evolving evidence-base for stroke prevention and management, there is a need for robust data and evidence to inform future policy decision-making. Population-based modelling and economic evaluation of alternative policy options is a useful tool to support decision making. However, this process must be aligned to key stakeholder priorities. The aim of the proposed research is to engage with stakeholders in Ireland to identify their priorities for the development of stroke prevention and management strategies and policies.

**Methods**

The design is iterative, based on mixed methods. Phase 1 involves a qualitative approach for initial priority gathering, based on an open-ended online survey (target sample: 100–120) and interviews (target sample: 34–40). Stakeholders will include: 1) stroke survivors and family member/main carers, 2) healthcare professionals (HCPs) providing stroke care and 3) people working in stroke research, policy and advocacy. These data will be analysed qualitatively, with the aim of identifying a long-list of specific interventions. Phase 2 involves an interim priority-setting exercise, based on a quantitative online survey. Participants will be asked to rank the interventions on the initial long-list. These rankings will be used to inform a final priority-setting workshop (Phase 3), where a small stakeholder group will decide on the final set of priorities.

**Discussion**

The rich and detailed quantitative and qualitative data, based on the views of diverse stakeholders, will be directly relevant to policy makers and service planners involved in developing and improving stroke care in Ireland. The information provided will also be essential to inform the Scenario and Intervention Modelling in Ireland for Stroke (SIMI-Stroke) project, a population-based economic and epidemiological modelling study aimed at identifying cost-effective interventions for stroke across the prevention, acute and post-acute care continuum.

## Introduction

Stroke is a major cause of death and disability globally ^1^. In 2017, 14% of stroke patients in Ireland died in hospital, 26% had moderate or severe disability post-stroke, and 34% of patients had mild disability ^2^. Approximately 20% of stroke survivors have dementia ^3^, with a further 40% having some level of cognitive impairment that does not meet the full criteria for dementia ^4^. Key advances in acute stroke treatment in recent years have resulted in improved survival ^5^, while better prevention and changing patterns of risk factors (e.g., reduced smoking) have led to reductions in age-specific stroke incidence ^5^.

Notwithstanding these significant advances, key challenges remain for stroke care delivery. Improved survival means greater numbers of people in need of rehabilitation and community services, an area that has been under-developed historically in Ireland ^6^. The number of people living with stroke in Ireland is projected to increase from 37,448 in 2016 to 69,051 (22 per 1000 population) in 2035 ^7^. In this context of more numerous stroke survivors, and a rapidly evolving evidence base for stroke prevention and management, with considerable potential to improve outcomes, there is a need for robust data and evidence to inform policy decision-making. This includes data on stakeholder priorities for service development, and on projected costs and outcomes associated with alternative policy options.

Population-based modelling and economic evaluation of alternative policy options is a key tool to support decision-making in relation to further development of stroke services in Ireland. The StrokeCog model has recently been developed for this purpose and can be used to evaluate the effectiveness and cost-effectiveness of alternative policy scenarios related to acute, post-acute, and prevention stroke services in Ireland ^7^. However, it is critical that this process of evaluating alternative intervention options is informed by the views and experience of those most affected - stroke survivors and their family members or main carers, and healthcare and other professionals involved in stroke care delivery.

The aim of the proposed research is to engage with stakeholders in Ireland to identify their priorities for the development of stroke prevention and management strategies and policies for Ireland. Stakeholders will include 1) stroke survivors and family member/main carers, 2) healthcare professionals (HCPs) providing stroke care and 3) people working in research, policy, and advocacy.

The process outlined in this protocol will result in the identification of five priority interventions. These will then be evaluated using the StrokeCog model, as part of a broader study: “Scenario and Intervention Modelling in Ireland for Stroke: evaluating the effect of alternative policy scenarios and interventions for stroke in Ireland on outcomes and costs” (SIMI-Stroke). This will provide data on the potential costs and benefits of these options, to inform future policy and service planning. By focussing on evaluating interventions that are considered priorities by key stakeholders, we will ensure that the SIMI-Stroke study is aligned to and consistent with views and needs of those most involved in and most affected by policy and service provision in this area.

## Protocol

### Study design

**Figure 1.**
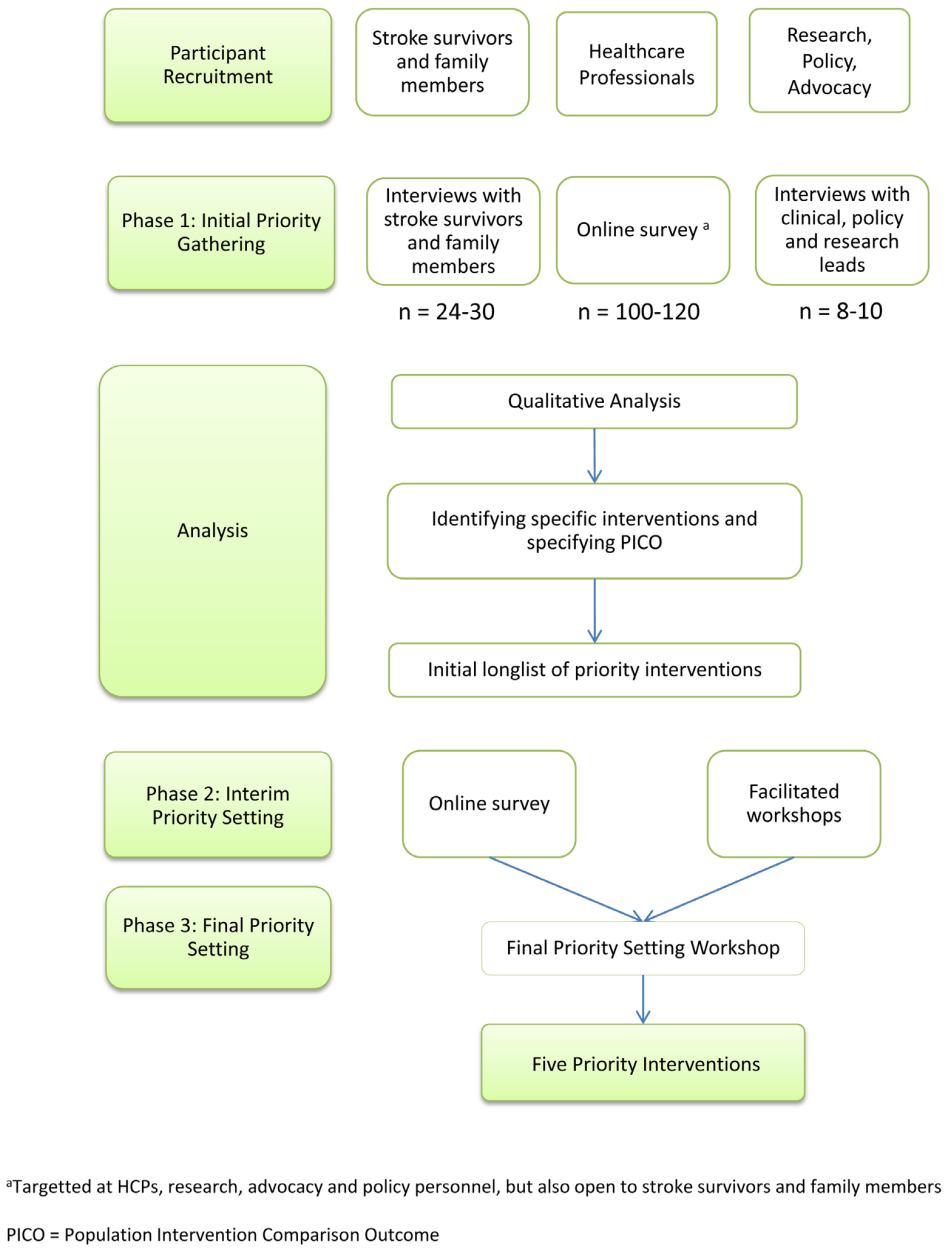
Research design for stakeholder engagement priority-setting study.

The research design is based on the method outlined by the James Lind Alliance (JLA) for identifying research priorities ^8^. The JLA guidance is intended for a full research priority-setting partnership exercise which is beyond the scope of this project, but the JLA methodology will be used as a guide. The design is iterative, based on mixed methods, with the phases outlined in Figure 1. Phase 1 will involve a qualitative approach for initial priority gathering, based on an open-ended online survey and interviews. These data will be analysed qualitatively, with the aim of identifying a long-list of specific priority interventions. Phase 2 will involve an interim priority setting exercise, based on a quantitative design. This will involve asking participants to rank the interventions on the initial long-list. These rankings will be used to inform a final priority-setting workshop (Phase 3), where a small group of stakeholders will decide on the final set of priorities. The JLA process typically identifies a list of ten priorities. Given the narrower scope of this topic, and the need to conduct an economic evaluation of each priority intervention, we will aim to identify the top five priority interventions.

### Participants

Three groups of stakeholders will be invited to participate: 1) stroke survivors and family members/main carers, 2) healthcare professionals (HCPs) providing stroke care and 3) people working in research, policy and advocacy related to stroke prevention and care in Ireland. The breadth of stakeholder engagement will help to ensure that the interventions evaluated as part of this research are based on the priorities of relevant stakeholders, and facilitate the exploration of where these priorities converge and differ across sub-groups.

*Phase 1 Recruitment:* Purposive sampling will be used. Stroke survivors and family members/main carers will be recruited through multiple sources of recruitment to ensure a heterogeneous group that is as reflective as possible of the stroke population. This will include recruiting through stroke support groups run by the voluntary organisation, the Irish Heart and Stroke Foundation (IHF), social media, other relevant voluntary bodies and patient organisations, and clinical settings (e.g., stroke outpatient clinics) where possible. We will aim to be as inclusive as possible in our recruitment, with accessible versions of recruitment materials available for individuals with cognitive or communication difficulties. These will include aphasia-friendly materials, based on templates available from the NIHR Stroke Research Network ^9^. Sample consent forms and participant information leaflets are provided in the *Extended data* ^10^. Anyone with the capacity to provide informed consent to participate in the study will be included (see Ethical considerations section below for more detail on the consent process).

HCPs and policy stakeholders will be recruited through existing networks of the research team and collaborators. Efforts will be made to recruit HCPs via hospital management, community health organisations, and professional organisations including (but not limited to) the Irish College of General Practitioners (ICGP), the Association of Occupational Therapists in Ireland (AOTI) and the Irish Society for Chartered Physiotherapists (ISCP). We will also recruit through social media, including designated Facebook and Twitter accounts for the project. For the HCPs, we will aim for a multidisciplinary mixed sample across primary, secondary and tertiary care. For all stakeholder groups, we will aim for geographical diversity, as service levels and needs may vary regionally.

We will aim to interview a minimum of 12–15 stroke survivors and 12–15 family members/main carers. For the online survey, we will aim for 100–120 responses in total, 40–50 from HCPs and 40–50 from people working in research, policy and advocacy, and a minimum of 20 responses from stroke patients and family members/main carers. The same recruitment method will be used for the interview and survey, as outlined above.

We will also carry out in-depth interviews with individuals identified as key clinical, policy and research leaders in stroke (8–10 interviews). These will be identified based on discussions with our collaborator network and review of publicly available information (e.g., websites, policy documents). It will include a mix of national leads of key policy programmes, balanced by senior clinicians working at a regional level. 

Based on the relatively narrow scope of the topic, we believe the target sample size will be sufficient to achieve saturation. Saturation in this study will be considered at two levels – code saturation (no additional issues identified) but also meaning saturation (no further dimensions, nuances or insights of issues identified) ^11^. As recruitment and data collection progresses, we will identify any gaps in the participant profile (e.g., age, geographical area, severity, specific patient/service experiences) and re-target recruitment strategies accordingly.

*Phase 2 recruitment:* The same stakeholders (HCPs, stroke patients and family, individuals working in policy, research and advocacy) who participated in the initial stage will be invited to participate again in an online ranking project. Each participant in Phase 1 will be asked to consent to further contact in relation to participation in this second phase. As not all phase 1 participants will also take part in phase 2, to achieve a similar sample size to phase 1 (n=135–160), we will also recruit further participants through the same channels as Phase 1 – social media, organisations such as the IHF, SCTNI, and professional bodies.

*Phase 3 Recruitment:* For final priority setting, approximately 12–15 stakeholders (balanced across stakeholder groups) will be invited to take part in a one-day workshop, as recommended by the JLA ^8^. Phase 1 and 2 participants who consented to further contact will be invited to take part in the workshop. If there are more participants interested than workshop spaces available, the participants will be chosen at random, again with a balance across stakeholder groups.

### Data collection and analysis

*Phase 1:* This phase involves two modes of data collection – qualitative interviews and an open-ended online survey, conducted in parallel. For survivor and main carer interviews, a semi-structured topic guide has been developed. Topics include: experience of the stroke itself, description of patient journey including acute and post-acute care; views on potential changes and improvements to services. A separate semi-structured topic guide has been developed for use with clinical, policy and research leaders, specifically tailored to this group. All interviews will be conducted by the lead author (ES), who has training and experience in qualitative data collection and analysis. Detailed field notes will also be recorded before and after the interviews to collect key contextual information. The topic guide (included in the *Extended data* ^10^) has been and will continue to be revised iteratively as data collection progresses. For participants with cognitive and communication problems, strategies will be used to support communication in an interview setting. These include sharing the interview questions in advance, using clear and simple wording and allowing the person space to respond. The approach will be tailored to each individual interviewee.

The online survey for initial priority gathering includes nine open-ended questions. These cover different areas of stroke care, for example hospital care, rehabilitation, information provision and stroke prevention. Different question wording is used depending on whether the respondent is a 1) stroke survivor, 2) family member/main carer or 3) HCP or people working in research, policy, advocacy.

The survey was piloted by a small number (n=4) of individual members of the target groups – two researchers, an allied health professional and a stroke survivor (our PPI representative). The pilot version included questions at the end for feedback, such as how long the survey took, whether the information and questions were clear and easy to understand, and whether the survey was easy to complete overall, with a combination of closed and free text responses. The feedback from the pilot was positive, with some useful comments resulting in minor revisions of the survey instructions. The length of time the survey took to complete was consistent with our initial estimates of 30–45 minutes. The online survey content is provided in the supplementary material ( *Extended data* ^10^).

All data collection materials were developed based on Donabedian’s three aspects of healthcare quality – structure, process and outcome ^12^, and informed by literature review. Current best practice guidelines were reviewed to identify aspects of stroke healthcare that are potentially important to healthcare professionals ^13– 16^, and previous qualitative literature was reviewed to identify aspects of stroke care potentially important to patients and family members/main carers ^17, 18^. Members of the relevant target stakeholder groups on the collaborator team, including our PPI collaborator, also reviewed and provided feedback on the topic guide and survey questions. 

At the project outset, we will focus on remote interviewing methods to ensure there is no COVID-19 contamination risk associated with data collection. However, as COVID-19 contamination risk levels and regulations change with the vaccine roll-out, there may be opportunities to carry out face-to-face interviews.

Interviews will be transcribed and responses to the open-ended online survey questions will be collated. These data will be analysed using framework analysis, broadly following the approach outlined by Gale *et al.* ^19^. Each interview transcript and individual survey response will be defined as a case. The first stage of analysis is familiarisation, involving a close reading of data related to a sub-set of cases, followed by coding, where an initial set of analytic codes and categories will be identified, with a focus on information that is relevant to the research question. This will facilitate the development of a working analytic framework, which can be applied to the remaining qualitative data, while also being refined in an iterative way. Following this, the data will be charted into a framework matrix, which will allow the data to be summarised for each case, by the categories in the analytic framework. The final stage of analysis is interpretation, which will involve identifying links and connections across categories, and comparing across cases. The framework method provides a systematic model for management of relatively large datasets and allows for comparison within and across cases, and is thus a suitable approach for the current study. NVivo (Version 12, QSR international) software will be used for data management and analysis. Once analysis is complete, NVivo files will be converted to an open format (e.g., .txt, .rtf).

The interpretation phase of analysis will also involve identifying specific interventions that relate to the categories identified within the analytic framework. This may include examples where a category can be clearly linked to a specific intervention, or instances where further interpretation is needed. For example, if a care need is identified for a specific phase post-stroke or a specific problem, without specifying an intervention, then a potentially relevant intervention that can be mapped to this phase of care or problem will be identified. For each specific intervention, a PICO will be developed - patient, intervention, comparison and outcome. These interpretations will be circulated to the collaborator team and patient representatives (including PPI representatives and the IHF advocacy team) for them to individually review, helping to ensure accuracy and fairness. The process will result in a long- list of potential priority interventions.

*Evidence for effectiveness:* Once the initial long-list has been identified, we will begin identifying evidence for the effectiveness of each intervention. For each intervention, we will first identify whether a Cochrane review exists; if no Cochrane review exists, we will then identify whether a systematic review has been published in a peer review journal; if no systematic review is identified, we will investigate whether a randomised controlled trial (RCT) has been published. Evidence from scoping reviews and descriptive reviews, and from observational studies, will also be identified where appropriate. This information will be used to inform final identification of interventions in phase 3.

*Phase 2:* The same stakeholders (HCPs, stroke patients and family, individuals working in policy, research and advocacy) who participated in the initial stage will be invited to participate again in an online ranking exercise or survey. This will consist of the initial long-list of priority interventions, which participants will be asked to rank in order of importance.

The JLA recommends several ranking formats, and we will use the simplest of these, which involves asking people to choose the five most important interventions. They will not be required to rank those five in order of importance. This simple approach is likely to be most inclusive for all participants.

Stroke survivors and family members/main carers will be invited to participate in the online exercise, but will also be given an option of facilitated live sessions, in individual or group format. The final format of the live facilitated sessions will be decided in consultation with patient representatives and relevant gatekeepers (e.g., IHF and stroke support group leaders), and may depend on COVID-19 risk levels and regulations. These sessions will not be recorded – the only data gathered will be the individual participant rankings, and high-level information on the stakeholder group (person who had a stroke or family member/main carer), and geographical region.

A simple, straightforward approach will be used to analyse the ranking data, following the JLA guidance ^8^. Each intervention will receive a point for each time it is chosen by any respondent. Totals for each intervention will be calculated by summing over all respondents for each by stakeholder group. This information will be presented at the Phase 3 workshop to inform identification of a final set of interventions.

*Phase 3:* Following the JLA guidance, the nominal group technique ^20^ will be used at the stakeholder workshop. This approach involves opportunities for each individual to give their views, structured small group discussions, and shared voting or ranking. It facilitates both quick decision-making and consideration of everyone’s opinion. The workshop will not be recorded, but we will gather and store documentation and notes from the session to ensure transparency. Individual contributions will not be identifiable.

We have pre-specified some criteria for the composition of the final set of interventions, to ensure a sufficient evidence base and balance across the stroke care continuum.

• At least 4/5 must have RCT evidence of effectiveness for at least one relevant outcome (survival, functional outcomes, cognitive outcomes)
• At least 3/5 must have systematic review RCT evidence for effectiveness for at least one relevant outcome

Therefore, an intervention with effectiveness evidence based on observational, non-RCT evidence could be included in the final set if it is highly ranked, but no more than one intervention with this standard of evidence will be included.

In addition, the following criteria will apply:

• At least 1/5 must be a primary prevention intervention
• At least 1/5 must be an acute intervention
• At least 1/5 must be a non-acute intervention (potentially including social care).

We therefore anticipate there will be a minimum of one intervention from each part of the stroke care continuum. The inclusion of at least one primary prevention intervention will ensure a balance between interventions focused on individual gains and population gains.

A discussion with PPI collaborators has emphasised the importance of including interventions with non-RCT evidence within the study, as some of the services seen as most valuable by stroke survivors (e.g., support to identify and co-ordinate relevant services, peer support groups) may not have RCT evidence available.

### Ethical considerations

Phase 1 of the study has been granted ethical approval from the RCSI Research Ethics Committee (REC) (approval number 202101017), and ethical approval will be sought from RCSI REC for subsequent phases.

It will be assumed that individuals have the capacity to consent, unless otherwise demonstrated, as per the Assisted Decision-Making Capacity Act (2015) and the National Consent Policy. Where appropriate, we will consult with gatekeepers (e.g., stroke support group co-ordinators) and people who have a close relationship with the individual to establish prospective participants’ likely capacity to consent, before we approach them, so that we can properly support any participants with possible diminished capacity.

The lead researcher (ES) will give prospective interview participants a brief overview of what participation entails. The participant information leaflet (PIL) outlining all relevant aspects of the study including the purpose and nature of the research, its consequences and risks, as well as information on the management of personal data, will be sent via email or post, along with a consent form. After approximately one week, ES will arrange a follow up call. During this call, ES will go over the PIL and consent form to ensure full understanding of the study aims and what participation involves. If the person decides to participate, they will return the signed consent form (written or digital signature) by email or post (using a pre-paid postage stamped addressed envelope). Once the signed consent form is received, a phone or video call interview will be scheduled at a time and format convenient to the participant.

Consent will be sought separately for audio recording and participants will be informed of their right to review transcripts. If participants seek to amend or add further details to transcripts, this will be recorded in field notes. Consent will also be assessed on a continual basis – following the initial informed consent, consent will again be checked prior to the interview, during the interview, and after the interview has concluded (i.e., using process consent to ensure the person is still happy to take part at all times).

Should participants become upset during the course of the interview the participant will immediately be offered the option of terminating the interview. Information on support services (e.g., the Irish Heart Foundation) will be provided and, following discussion with the participant, a family member/main carer or GP will be informed, where relevant.

The online survey will be accessed anonymously via an online platform. Participants must indicate that they have reviewed the participant information and consent to participate before being able to respond. 

A data protection impact assessment (DPIA) has also been undertaken for Phase 1 data collection and processing activities, in consultation with the RCSI Data Protection Officer. Interview transcripts and qualitative survey responses will be pseudonymised, with quantitative survey data fully and irrevocably anonymized on survey completion. Personal identifiable data (e.g., contact details), will only be stored with explicit consent and for a specific purpose (e.g., contacting the person about the research). The DPIA also includes a map of the project data flows, to ensure transparency and GDPR compliance at all stages of data collection, processing, storage and destruction.

Data collection will be limited to what is adequate and relevant to the purpose of the research project (GDPR 'data minimisation' principle). Appropriate technical and organisational measures are in place to protect against unlawful or unauthorised processing, as well as accidental loss or destruction. Personal data will be stored securely on the RCSI server, in a password-protected folder accessible only to named personnel. Personal data that are no longer required will be deleted or disposed of in a secure manner.

### PPI involvement

A PPI representative will sit on the overall study steering group to ensure inclusion of the patient perspective in the overall guiding of the research. Separate one-to-one meetings will also be scheduled (one or two per year) to facilitate more in-depth updates and feedback.

### Dissemination and FAIR data

Results will be submitted for publication in a peer-reviewed journal. We will also produce a lay summary and a policy brief for dissemination through relevant stakeholder networks. Key findings will be presented at national and international conferences. A data management plan has been produced for the project, in consultation with a data specialist, to ensure that FAIR data principles are adhered to (i.e., that data is Findable, Accessible, Interoperable, Reusable). Raw qualitative transcripts and open-ended survey responses will not be shared to ensure protection of personal identifiable data, but detailed documentation related to data collection and analysis (e.g., audit trails) will be available in appendices of published papers.

### Reporting

Publications describing the qualitative data will follow the Consolidated criteria for reporting qualitative research (COREQ) checklist ^21^. Reporting of quantitative survey data will following the STROBE guidelines ^22^.

### Study status

Recruitment and data collection for Phase 1 commenced in April 2021.

## Discussion

The study will provide important evidence in relation to stakeholder priorities for improving and developing stroke care in Ireland. The qualitative data generated will provide rich and detailed evidence in relation to the areas of stroke health care that are working well. It will also help to identify areas that are in need of improvement, for example where needed services are difficult to access, or where there are unmet needs or gaps in the services provided. The data from the ranking survey and stakeholder workshop will provide specific information as to which areas of development should be prioritised.

This information will be useful for orienting policy and service planning in and of itself, and will be critical to inform further research as part of the SIMI-Stroke project. The current over-arching policy for health services in Ireland, Sláintecare, emphasises the need for population health-based planning to inform service decisions ^23^. This population-based approach is exemplified by the SIMI-Stroke project, which will draw on multiple data sources to create a population-based simulation of stroke disease progression in the Irish population, and apply evidence on specific intervention effectiveness to improve health outcomes. By engaging with stakeholders from the project outset, we will ensure that the research is aligned with their priorities, and maximise the potential for findings to be implemented and translated into benefits for stroke survivors. 

**Figure 2.**
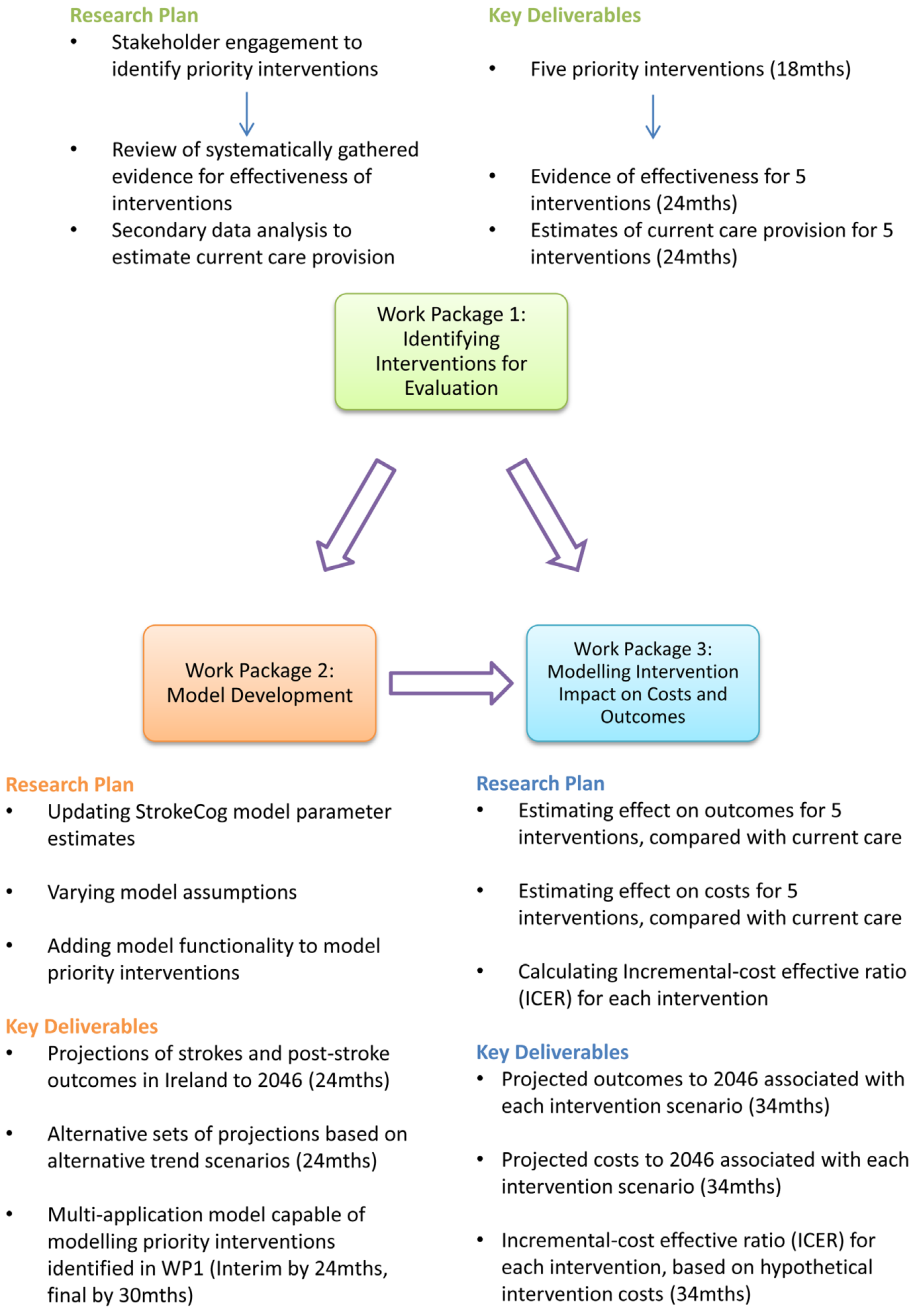
Research plan and objectives SIMI-stroke research project.

The SIMI-Stroke project involves three inter-related work packages, outlined in Figure 2. Work package 2 (WP2) will involve further development of an already existing epidemiological and economic model that can be applied to predict the potential impact of the priority interventions identified in WP1. In WP3, the model will be applied to these priority interventions to predict potential future costs and outcomes in the Irish population.

### Limitations

As outlined previously, it will be important to ensure that the final set of interventions to be evaluated have sufficient supporting evidence for effectiveness, to maximise relevance for policy making and potential for implementation. If an intervention with effectiveness evidence based on observational, non-RCT evidence is highly ranked by stakeholders, it could be included; however, only one such intervention will be included in the final set. This emphasis on the standard of evidence may lead to a bias towards acute care interventions which are more likely to have RCT-level evidence. We will therefore also aim to identify and highlight where a mismatch exists between the volume of research related to a specific intervention or phase of care, and its value and relevance to stakeholders, particularly survivors and carers.

Another potential limitation relates to the feasibility of the target sample size (100–120) for the Phase 1 qualitative survey, which may be ambitious given the length of time required to complete the survey. It is possible that a smaller sample will be more feasible and sufficient for saturation, depending on the depth and detail of information collected.

## Conclusion

There is a particular need for modelling in stroke as the evidence-base is rapidly evolving, with new treatments coming on-stream and requiring the development of new services and models of care. In addition, improved survival leads to an increased need for rehabilitation and community services. The SIMI-Stroke project will produce a unique resource for planning and decision-making in stroke prevention and management in Ireland, which is not currently available. The stakeholder engagement study outlined here will ensure that SIMI-Stroke is guided by stakeholder priorities, and provides rich contextual information, based on experiential knowledge, that will complement the quantitative data generated through epidemiological modelling. Explicit involvement of stakeholders from the project outset will also help to maximise the uptake and implementation of study recommendations and findings.

## Data availability statement

### Underlying data

No data are associated with this article.

### Extended data

Open Science Framework: Identifying priority interventions for stroke in Ireland through stakeholder engagement to inform population-based modelling: a mixed methods protocol - supplementary material, https://doi.org/10.17605/OSF.IO/QJGUZ ^10^

This project contains the following extended data:

- Participant Information Leaflet for stroke survivors
- Participant Information Leaflet for stroke survivors – aphasia-friendly
- Consent form for stroke survivors
- Semi-structured interview topic guide for stroke survivors
- Online survey – participant information and survey questions

Data are available under the terms of the Creative Commons Attribution 4.0 International license (CC-BY 4.0).

## References

[1] GBD 2015 Neurological Disorders Collaborator Group: Global, regional, and national burden of neurological disorders during 1990–2015: a systematic analysis for the Global Burden of Disease Study 2015. *Lancet Neurol.* 2017;16(11):877–97. 10.1016/S1474-4422(17)30299-5 28931491 PMC5641502

[2] National Stroke Programme: National Stroke Register Report 2017.2018. Reference Source

[3] Pendlebury ST Rothwell PM : Prevalence, incidence, and factors associated with pre-stroke and post-stroke dementia: a systematic review and meta-analysis. *Lancet Neurol.* 2009;8(11):1006–18. 10.1016/S1474-4422(09)70236-4 19782001

[4] Sexton E McLoughlin A Williams DJ : Systematic review and meta-analysis of the prevalence of cognitive impairment no dementia in the first year post-stroke. *Eur Stroke J.* 2019;4(2):160–71. 10.1177/2396987318825484 31259264 PMC6591758

[5] Seminog OO Scarborough P Wright L : Determinants of the decline in mortality from acute stroke in England: linked national database study of 795 869 adults. *BMJ.* 2019;365: l1778. 10.1136/bmj.l1778 31122927 PMC6529851

[6] McElwaine P McCormack J Harbison J : Irish Heart Foundation/HSE National Stroke Audit Rehabilitation Units 2016. Vol. October.2016. Reference Source

[7] Sexton E Donnelly NA Merriman NA : StrokeCog Markov Model: Projected Prevalent and Incident Cases of Stroke and Poststroke Cognitive Impairment to 2035 in Ireland. *Stroke.* 2021; STROKEAHA121034005. 10.1161/STROKEAHA.121.034005 34496624

[8] The James Lind Alliance: The James Lind Alliance Guidebook. Version 8.2018. Reference Source

[9] NIHR Stroke Research Network: Templates for accessible information sheets and consent forms. [cited 2021 Aug 20]. Reference Source

[10] Sexton E : Identifying priority interventions for stroke in Ireland through stakeholder engagement to inform population-based modelling: a mixed methods protocol - supplementary material.2021. 10.17605/OSF.IO/QJGUZ PMC10985459 38567097

[11] Hennink MM Kaiser BN Marconi VC : Code Saturation Versus Meaning Saturation: How Many Interviews Are Enough? *Qual Health Res.* 2017;27(4):591–608. 10.1177/1049732316665344 27670770 PMC9359070

[12] Donabedian A : The Quality of Care. How Can It Be Assessed? *JAMA.* 1988;260(12):1743–8. 10.1001/jama.1988.03410120089033 3045356

[13] European Stroke Organisation (ESO): ESO Guideline Directory. [cited 2021 Jan 14]. Reference Source

[14] Intercollegiate Stroke Working Party: National Clinical Guideline for Stroke.2016. Reference Source

[15] Mountain A Lindsay MP Teasell R : Canadian Stroke Best Practice Recommendations: Rehabilitation, Recovery, and Community Participation following Stroke. Part Two: Transitions and Community Participation Following Stroke. *Int J Stroke.* 2020;15(7):789–806. 10.1177/1747493019897847 31983292

[16] Norrving B Barrick J Davalos A : Action Plan for Stroke in Europe 2018–2030. *Eur Stroke J.* 2018;3(4):309–336. 10.1177/2396987318808719 31236480 PMC6571507

[17] Pindus DM Mullis R Lim L : Stroke survivors’ and informal caregivers’ experiences of primary care and community healthcare services – A systematic review and meta-ethnography. *PLoS One.* 2018;13(2):e0192533. 10.1371/journal.pone.0192533 29466383 PMC5821463

[18] McKevitt C Redfern J Mold F : Qualitative studies of stroke: a systematic review. *Stroke.* 2004;35(6):1499–505. 10.1161/01.STR.0000127532.64840.36 15105517

[19] Gale N Heath G Cameron E : Using the framework method for the analysis of qualitative data in multi-disciplinary health research. *BMC Med Res Methodol.* 2013;13(117):1–8. 10.1186/1471-2288-13-117 24047204 PMC3848812

[20] Manera K Hanson CS Gutman T : Consensus Methods: Nominal Group Technique. In: Liamputtong P, editor. *Handbook of Research Methods in Health Social Sciences*. Singapore: Springer;2019;737–50. 10.1007/978-981-10-5251-4_100

[21] Tong A Sainsbury P Craig J : Consolidated criteria for reporting qualitative research (COREQ): a 32-item checklist for interviews and focus groups. *Int J Qual Health Care.* 2007;19(6):349–57. 10.1093/intqhc/mzm042 17872937

[22] von Elm E Altman DG Egger M : The Strengthening the Reporting of Observational Studies in Epidemiology (STROBE) Statement: guidelines for reporting observational studies. *Int J Surg.* 2014;12(12):1495–9. 10.1016/j.ijsu.2014.07.013 25046131

[23] Committee on the Future of Healthcare. Sláintecare Report.2017;191. Reference Source

