# Supplementary material for: Identifying priority interventions for stroke in Ireland through stakeholder engagement to inform population-based modelling: a mixed methods protocol
Source: HRB Open Res. 2021 Sep 30;4:109. [Version 1] doi: 10.12688/hrbopenres.13413.1 (PMC10985459; doi:10.12688/hrbopenres.13413.1)
Supplement: Peer review history

# Reviewer report: Reviewer response for version 1

**DOI:** 10.21956/hrbopenres.14613.r38490

**Published:** 2024-04-09

**Authors:** Rosalie McDonough

**Affiliations:** 1 University of Calgary, Calgary, Alberta, Canada

With this protocol, the authors present a comprehensive and structured approach to engage stakeholders in the development of stroke prevention and management strategies and policies for Ireland. The protocol outlines a three-phase research design, incorporating qualitative interviews, online surveys, and a final priority-setting workshop. 

 This protocol presents a well thought out and comprehensive approach to engaging stakeholders in shaping stroke prevention and management strategies in Ireland. The structured methodology, including qualitative interviews and online surveys, ensures diverse perspectives are captured. The emphasis on ethical considerations and patient and public involvement enhances the study's credibility and relevance. Overall, this protocol will provide important insights into stakeholder priorities, facilitating evidence-based decision-making in stroke care.

 I am excited to read the results. And perhaps a road map for other countries of diverse/larger geographies who may be looking to do the same.

Is the study design appropriate for the research question?

Yes

Is the rationale for, and objectives of, the study clearly described?

Yes

Are sufficient details of the methods provided to allow replication by others?

Yes

Are the datasets clearly presented in a useable and accessible format?

Not applicable

Reviewer Expertise:

Neuroradiology, CT, MRI, EVT patient selection, acute ischemic stroke

I confirm that I have read this submission and believe that I have an appropriate level of expertise to confirm that it is of an acceptable scientific standard.

# Reviewer report: Reviewer response for version 1

**DOI:** 10.21956/hrbopenres.14613.r38491

**Published:** 2024-04-01

**Authors:** Annemarei Ranta

**Affiliations:** 1 Medicine, University of Otago, Dunedin, Otago, New Zealand

This protocol describes a mixed methods study aiming to set stroke service development priorities though stake holder engagement. The project is worthwhile and the paper well written.

 I just have a few comments for the authors to consider:

 (1) Why do you need to prioritise interventions supported by RCT data?  Shouldn't these be implemented regardless?  Do you not have a national guideline?  I presume this is due to resource constraints and it would be worth mentioning this explicitly.

 (2) Given you will focus on RCT based interventions would it be useful to identify those first and present these to stakeholders to choose from?  Just wondering if that might be more efficient?

 (3) Have you considered a realist evaluation?  This would take into consideration context - might be beyond the scope of this study.

 (4) I wondered why you have only 1/3 consumers.  maybe 50/50 would be better to be able to call it 'co-design.?' Also will you be separating responses by group so when you review prioritizations you can take into account who favored what?  there may be differences between stake holder groups which may be important to note.

 (5) Will consumers get raining in priority setting?

 (6) Will geography be considered when assessing results?  Just thinking that one geographic area may have quite different needs than another.  In fact I was a bit unclear where this would take place - does it involve all of Ireland?  I would have thought that needs/priorities might differ quite a bit by locations e.g. urban versus rural?

Is the study design appropriate for the research question?

Yes

Is the rationale for, and objectives of, the study clearly described?

Yes

Are sufficient details of the methods provided to allow replication by others?

Yes

Are the datasets clearly presented in a useable and accessible format?

Yes

Reviewer Expertise:

Health services research in stroke care

I confirm that I have read this submission and believe that I have an appropriate level of expertise to confirm that it is of an acceptable scientific standard.

# Reviewer report: Reviewer response for version 1

**DOI:** 10.21956/hrbopenres.14613.r30561

**Published:** 2021-12-16

**Authors:** Eirini Kontou

**Affiliations:** 1 School of Medicine, University of Nottingham, Nottingham, UK; 2 Nottinghamshire Healthcare NHS Foundation Trust, Nottingham, UK

This is a study protocol using a mixed-methods approach to engage with stakeholders for identifying priority interventions for stroke in Ireland. This seems a well-designed project in a field where more research is needed. It describes three phases and the design is iterative including quantitative and qualitative methods.

 It seems that this is a protocol for a study that is now underway (on status noted that recruitment and data collection for Phase 1 commenced). Comments are focused on those aspects that perhaps need clarifications and are still in progress. I hope this would be helpful to the authors when they publish their work as a scientific paper.

- The introduction is clear and adequately covers the background and purpose. Perhaps, the authors could have included a bit more information on the StrokeCog model as it seems key for this work and the broader study (SIMI-Stroke) that aims to evaluate the identified priority interventions. The aim is stated at the end but maybe a section on ‘Aims’ can outline a series of secondary objectives for each phase.
- The impact of stroke can vary considerably and the authors acknowledge the need to recruit participants through multiple sources to ensure a ‘ *heterogeneous group that is as reflective as possible of the stroke population*’. It would be useful to know a little more about how stroke survivors with severe cognitive or communication impairments will participate if they do not have the capacity to consent.
- The protocol sections cover detailed information on study design, participants, data collection, and analyses. PPI input is highlighted across the protocol, but the section on PPI involvement is rather brief and limited to only two sentences.
- Ethical approval has been granted and considerations discussed. Consent procedures are highlighted, however, a process for consent by proxy does not seem to be included here. This can be particularly relevant for stroke survivors with cognitive and communication difficulties who may require further support with the completion of the study documentation.
- In Phase 3, the nominal group technique will be used and would be helpful to include some references to justify this decision. Approximately 12-15 stakeholders will be included and authors state that will balance representation for each stakeholder group. Perhaps you can include some further details on how this balance will be achieved.
- Recruitment methods and approaches to maximise response rates are described. In addition to the target sample size for each phase, it would be useful to provide some justification on the numbers provided (for instance, references of stroke studies or national priority settings). In the limitations, it is mentioned that sample size for surveys may be more difficult to attain so would be desirable to consider at what response rate you would consider the surveys to have been a success. Also, how will you ensure and record response rates for sufficient representation from each participant group, recruitment source, or organisation?
- The ongoing impact of COVID-19 on the overall project and relevant mitigations have been considered. In this case, it is useful to pre-specify some criteria around recruitment, etc. Assuming that the project is ongoing, there would be an opportunity to add these to this protocol.

In summary, this is a well-written protocol that describes the methods for an ambitious project that will inform future work for stroke health services in Ireland.

Is the study design appropriate for the research question?

Yes

Is the rationale for, and objectives of, the study clearly described?

Yes

Are sufficient details of the methods provided to allow replication by others?

Yes

Are the datasets clearly presented in a useable and accessible format?

Not applicable

Reviewer Expertise:

Stroke care, rehabilitation, clinical trials, development and evaluation of complex interventions.

I confirm that I have read this submission and believe that I have an appropriate level of expertise to confirm that it is of an acceptable scientific standard.
